# Temporal induction of pro-inflammatory and regulatory cytokines in human peripheral blood mononuclear cells by *Campylobacter jejuni* and *Campylobacter coli*

**DOI:** 10.1371/journal.pone.0171350

**Published:** 2017-02-14

**Authors:** Eman Hamza, Sonja Kittl, Peter Kuhnert

**Affiliations:** 1 Institute of Veterinary Bacteriology, Vetsuisse Faculty, University of Bern, Bern, Switzerland; 2 Department of Zoonoses, Faculty of Veterinary Medicine, Cairo University, Cairo, Egypt; Iowa State University, UNITED STATES

## Abstract

*Campylobacter jejuni* along with *C*. *coli* are major cause of human gastroenteritis worldwide. So far, the human immune response against *Campylobacter* is not entirely clear. We hypothesize that it is coordinated by an interaction between pro-inflammatory and regulatory cytokines which is influenced by bacterial and host-individual differences. Accordingly, we used peripheral blood mononuclear cells (PBMC) from healthy donors to study the primary systemic immune response to *C*. *jejuni* and *C*. *coli*. PBMC were stimulated by different strains of *C*. *jejuni* and *C*. *coli* for three time points (5, 10, 24 hours). The production of the pro-inflammatory (IL-6, IL-8, IFN-γ) and the regulatory (IL-10) cytokines were measured by ELISA. All strains induced higher levels of IL-8 and IL-6 than IFN-γ and IL-10. In contrast to IL-8 and IL-6, IL-10 showed a steeper increase over time. While IFN-γ did not show any further increase between 10 and 24 hours. Interestingly, there was a significant correlation between IL-8 and IL-10 which peaked at 24 hours. Despite the variability of the used bacterial strains, their effect on cytokine production was less pronounced than the inter-person differences. The strongest significant effect of the strain was on the level of IL-10. IL-10 and IL-6 were significantly influenced by strain-person interaction. In conclusion, the systemic immune response to *C*. *coli* and *C*. *jejuni* is characterized by an early pro-inflammatory reaction with later initiation of regulatory immune response which is influenced mainly by the host, explaining the individual variations in disease severity. Additional work is needed to determine the cellular sources of the produced cytokines as well as the campylobacter molecules that might contribute to this stimulation.

## Introduction

Campylobacteriosis is the most common foodborne zoonosis in Europe with chicken constituting the main reservoir [1]. Most outbreaks in humans are related to chicken food or water-borne sources [2]. Humans can get infected with multiple *Campylobacter* species, while the most pathogenic are *C*. *jejuni* and *C*. *coli* causing gastroenteritis [3]. However, the illness is generally mild, some individuals develop severe post infectious sequelae such as irritable bowel syndrome, reactive arthritis and Guillain-Barré syndrome [4].

The properties of the infecting strain and the host immune status are thought to be involved in the disease development [2]. Epidemiological studies showed differences in susceptibility of individuals to *Campylobacter*. Some patient groups are more susceptible like very young children, elderly people or those who suffer from defects in humoral or cell mediated immunity [5]. Such patients experience severe fatal clinical symptoms and are more likely to develop chronic carrier status and recurrent infection [6].

Despite the high prevalence of campylobacteriosis, it is still unclear how exactly the organism causes disease in humans. Several studies have shown that *Campylobacter* is able to induce acute inflammatory enteritis [7–9], pointing to a possible role of polymorphonuclear leucocytes [6]. *In vitro* experiments on a range of human-derived cell lines [8, 10, 11] have shown that *C*. *jejuni* induce IL-8 which have also been reported in stools of patients with campylobacteriosis [12]. IL-8 is one of the earliest pro-inflammatory cytokines that are induced by enteric bacteria [13]. Moreover, secretion of IL-6 following stimulation by *C*. *jejuni* [8, 9, 14] and *C*. *coli* was also reported [15]. Beside its inflammatory action, IL-6 plays a critical role in governing the transition from innate to acquired immunity [13]. An *in vitro* model of *C*. *jejuni* infection using healthy human gut explant showed marked induction of IFN-γ with a modest increase of IL-22 and IL-17A levels [16]. IFN-γ was also shown to be associated with protection from campylobacteriosis [4]. Furthermore, *C*. *jejuni* was shown to induce the production of various pro-inflammatory cytokines as well as the regulatory cytokine IL-10 by dendritic cells [9].

Our hypothesis was that the immune response against *Campylobacter* is determined by a balance between pro-inflammatory and regulatory cytokines which is influenced by bacterial strains as well as the host immune system. Disturbance in this balance might drive the host immune response from mild to severe disease.

So far, most of the studies that examined the human immune response to *Campylobacter* used either cell line models [8, 10, 17] or human gut epithelial cells [10, 18]. The drawback of using cell lines is that it does not always reflect the natural reaction [11, 19]. Using primary tissue explant represents the gold standard, however, it raises ethical questions. In contrast, peripheral blood mononuclear cells (PBMC) can easily be obtained and have been used as a model to determine the cytokine response induced by enteric bacteria [20]. *C*. *jejuni* was shown to invade the colonic mucosa [16, 19, 21], suggesting an interaction with immune cells in intestinal mucosal compartments. Although PBMC cannot fully represent the immune cells in the intestinal mucosa, they share phenotypical similarities like the pattern recognition receptors on lymphocytes and macrophages [13] which could constitute a link between both populations. Moreover, *C*. *jejuni* was shown to persist within peripheral blood monocytes for up to 7 days [22]. Therefore, examination of the immune-stimulatory effect of *C*. *jejuni* and *C*. *coli* on PBMC can provide important indications of the mucosal immune response to these bacteria. Accordingly, the present study aimed to compare the production of pro-inflammatory (IL-8, IL-6 and IFN-γ) and regulatory (IL-10) cytokines by human PBMC following *in vitro* stimulation with various strains of *C*. *jejuni* and *C*. *coli* and examine whether there are inter-strain or inter-person differences in the immune response against *Campylobacter*.

## Materials and methods

### Study group

Blood samples from ten clinically healthy human volunteers were obtained by venepuncture and collected into sterile sodium heparin containing vacuette tubes (Greiner Bio-One vacuette GmbH). The group included five men and five women with a mean age of 43, range (24–64 years). Ethical clearance to use human subjects was obtained from the designated health facility. Written consent was obtained from each person upon information of the use of blood samples. The biological material was used anonymized. The study was approved by the institutional ethics committee at the Department of Pathobiology and Infectious Disease, University of Bern, and complied with the Declaration of Helsinki.

### Bacterial strains

Six genotypically characterized campylobacter strains were used. Two were *C*. *coli* of the same genotype, one chicken (Mon2065: ST-2142, fla66) and one human clinical isolate (N08-1636: ST-2142, fla66). Four strains were *C*. *jejuni*, three chicken isolates (Mon1296: ST-45, fla307; Mon1354: ST-45, fla307 and Mon227: ST-45, fla66) and one human isolate from a sepsis case (BK: ST-572, fla96). The bacteria were grown on tryptone soya agar plates with sheep blood (Oxoid) at 42°C under microaerophilic conditions for 24h, then harvested by washing with sterile phosphate buffered saline (PBS, GIBCO®) and centrifugation at 10°C for 10min at 4000g. This was followed by washing and subsequent re-suspension in PBS to an optical density of 1.0 McFarland. The bacteria were then diluted 1:200 in cell culture medium and subsequently used in the stimulation experiments.

### Isolation and stimulation of human PBMC

PBMC were isolated from whole blood using Ficoll (1077, GE Healthcare) gradient centrifugation. Equal volumes of blood and PBS-0.3mM EDTA were mixed and layered over Ficoll solution. After centrifugation (1260 g, 30 min), PBMC were collected from the interface, washed twice with PBS and re-suspended in cell culture medium to a final concentration of 1x10^6^ cells / ml. PBMC were seeded in duplicates into 24 well tissue culture plates and incubated with the same volume of each of the six bacterial suspensions with multiplicity of infection (MOI) 1.5 or lipopolysaccharide as a positive control (LPS, *E*. *coli* serotype O55-B5, 1μg/ml, Sigma-Aldrich, Buchs, Switzerland) or culture medium alone (mock) for 5, 10 and 24 hours at 37°C. Cell culture supernatants were collected, filtered through 0.2 μm syringe filters (Acrodisc®, Pall Corporation) and kept at -80°C until cytokine analysis using enzyme-linked immunosorbent assay (ELISA).

All cell cultures were performed in RPMI 1640 containing 10% native autologous human serum, nonessential amino acids (1%), MEM vitamins (100μM), sodium pyruvate (1mM) and gentamicin (25 μg/ml) to prevent bacterial overgrowth.

### ELISA

IL-6, IL-8, IL-10 and IFN-γ in cell culture supernatants were determined using sandwich ELISA kits (PeliKine compact^TM^, Sanquin) according to the manufacturer’s instructions. The samples were measured in triplicates, a standard curve of the relevant recombinant protein was included to calculate cytokine concentrations in the cell culture supernatant using SOFTmax program (SOFTmax® PRO, version 3.1.2, Molecular Devices).

### Statistical analysis

The NCSS 9 software program was used for statistical analyses. Descriptive statistics were run on the data and Kolmogrov–Smirnov test showed that the data were not normally distributed. Therefore, the data were transformed using natural logarithm.

**Fig 1:**
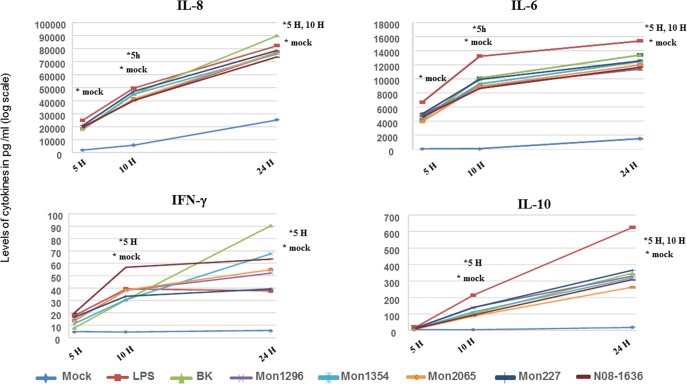
Induction of pro-inflammatory and regulatory cytokines by human PBMC following stimulation. Freshly isolated peripheral blood mononuclear cells (PBMC) from ten donors were left unstimulated (mock), stimulated with LPS, four *C*. *jejuni* strains [human (BK) and chicken (Mon1296, Mon1354 and Mon227) isolates] or two *C*. *coli* [human (N08-1636) and chicken (Mon2065) isolates] for three time points (5, 10 and 24 hours). The concentrations (pg/ml) of IL-8, IL-6, IFN-γ and IL-10 were measured in cell culture supernatants using ELISA. Each data point represents mean values for each type of stimulation. Comparisons between the different stimuli as well as between the three time points were performed using Bonferroni pairwise multiple comparison. The sign *mock indicates significant differences between mock and all other stimuli. The signs *5h and *10h indicate a significant differences to the time point 5 hours and 10 hours, respectively. The asterisks indicate statistical significant differences of p ≤ 0.05.

**Fig 2:**
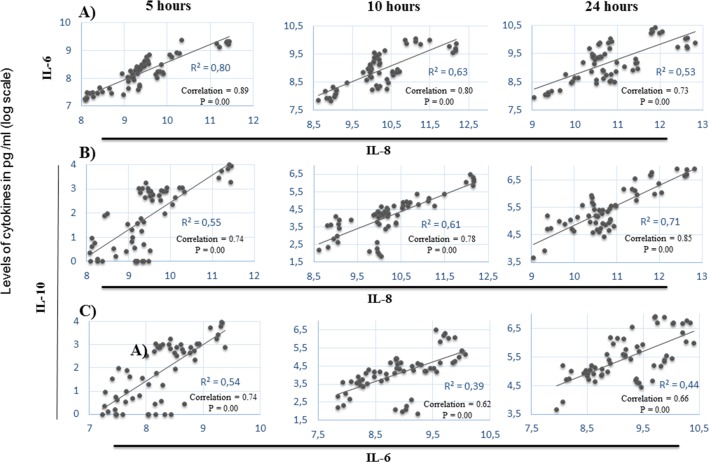
Correlation between the levels of IL-8, IL-6 and IL-10 following stimulation with the six strains of *Campylobacter*. Pearson Correlation coefficient was used to examine the correlation between the levels of IL-8 and IL-6 (A), IL-8 and IL-10 (B) and IL-6 and IL-10 (C). Each data point represents stimulation with one strain of *Campylobacter*. P values ≤ 0.05 were considered significant.

Balanced design analysis of variance MANOVA was used to examine the influence as well as the interaction of the factors “Person” [10 persons], “*Campylobacter* strain” [*C*. *coli* (Mon2065 and N08-1636); *C*. *jejuni* (Mon1296, Mon1354, Mon227 and BK)] and “Time point of incubation” [5, 10 and 24 hours] on the production of cytokines **(Table 1)**. **Table 1** Factors influencing the production of pro-inflammatory as well as regulatory cytokines by PBMC stimulated with different strains of *Campylobacter jejuni* and *C*. *coli*. Two statistical models (a, b) were used to examine the effect of person (Person), *Campylobacter* strains (Strains) and time of incubation (Time point) of the strains with PBMC on the cytokine production (Parameters). Two MANOVA models were run with the levels of cytokines as outcome variables and “Strains” as fixed factor **(Table 1)**. “Time point” was then set in one model **(Table 1A)** as the random factor with “Person” as fixed factor. While, in the other model **(Table 1B)**, “Time point” was the fixed factor and “Person” was the random factor. This was done to allow the analysis of the effect of all three parameters and also test for interactions. For significant factors in MANOVA, a pairwise multiple comparison with a Bonferroni correction of the significant level was used to determine significant differences in the level of cytokines between the different time points as well as bacterial strains and controls (mock, LPS) **(Fig 1)**. For comparison between time points within stimuli the Bonferroni multiple comparison test for two factor interaction was applied. Correlation between levels of cytokines for each time point was determined using Pearson correlation coefficient **(Fig 2)**.

**Table 1:** Factors influencing the production of pro-inflammatory as well as regulatory cytokines by PBMC stimulated with different strains of *Campylobacter jejuni* and *C*. coli*.

**a)** Person and Strains were set as fixed variables, while Time point was set as random variable.			
Cytokine		Factors		
		Person	Strains	Interaction Person / Strains
IL-8	**Sig (0.000)**	n.sig	n.sig
IL-6	**Sig (0.000)**	n.sig	n.sig
IL-10	**Sig (0.000)**	**Sig (0.008)**	**Sig (0.001)**
IFN-γ	**Sig (0.000)**	n.sig	**Sig (0.000)**
**b)** Time point and Strains were set as fixed variables, while person was the random variable.			
Cytokine	Factors		
	Time point	Strains	Interaction Time point / Strains
IL-8	**Sig (0.000)**	**Sig.(0.013)**	n.sig
IL-6	**Sig (0.000)**	**Sig.(0.001)**	**Sig (0.005)**
IL-10	**Sig (0.000)**	**Sig. (0.005)**	n.sig
IFN-γ	**Sig (0.000)**	n.sig	**Sig (0.000)**

* Two statistical models (a, b) were used to examine the effect of person (Person), *Campylobacter* strains (Strains) and time of incubation (Time point) of the strains with PBMC on the cytokine production (Parameters). Significant p-values are indicated in brackets.

Sig. means statistical significance of p ≤ 0.05. n.sig means not significant.

On each occasion, p values ≤ 0.05 were regarded as significant.

## Results

### Cytokines induction show significant inter-person variations and is influenced by person-*Campylobacter* strain interaction

PBMC from ten healthy donors were stimulated *in vitro* with six different *Campylobacter* strains of chicken and human sources, LPS as a positive control or left without stimulation (mock). The levels of pro-inflammatory (IL-8, IL-6, IFN-γ) and regulatory (IL-10) cytokines were measured in PBMC culture supernatants using ELISA. MANOVA was used to determine whether the cytokine production is influenced by the individual person, *Campylobacter* strains or post-stimulation time point **(Table 1)**.

There were no marked differences in the cytokine production between *C*. *jeuni* and *C*. *coli*, human and chicken isolates, or the genotypes of the strains. In contrast, the variables “Person” **(Table 1A)** as well as “Time point” **(Table 1B)** had a significant effect on the production of IL-8, IL-6, IFN-γ as well as IL-10. The variable “Strains" had a significant influence on IL-10 regardless of the model used **(Table 1A and 1B)**, while, IL-8 and IL-6 were significantly influenced by the “Strains” only when “Person” was set as a random variable **(Table 1B)**. Conversely, there was no significant influence of the “Strains” on the production of IFN-γ **(Table 1A and 1B)**. Interestingly, the interaction between strains and person “Person/Strains” **(Table 1A)** had a significant effect on IL-10 and IFN-γ, while the interaction between strains and time point “Time point/Strains” had a significant effect on IL-6 and IFN-γ **(Table 1B)**.

### All *Campylobacter* strains induce an early pro-inflammatory response followed by IL-10 in human PBMC

Comparing the production of cytokines between mock and *Campylobacter* stimulated PBMC showed a significant elevation of IL-8, IL-6, IFN-γ and IL-10 as compared to mock **(Fig 1)**. Freshly isolated peripheral blood mononuclear cells (PBMC) from ten donors were left unstimulated (mock), stimulated with LPS, four *C*. *jejuni* strains [human (BK) and chicken (Mon1296, Mon1354 and Mon227) isolates] or two *C*. *coli* [human (N08-1636) and chicken (Mon2065) isolates] for three time points (5, 10 and 24 hours). The concentrations (pg/ml) of IL-8, IL-6, IFN-γ and IL-10 were measured in cell culture supernatants using ELISA. Each data point represents mean values for each type of stimulation. Comparisons between the different stimuli as well as between the three time points were performed using Bonferroni pairwise multiple comparison. The sign *mock indicates significant differences between mock and all other stimuli. The signs *5h and *10h indicate a significant differences to the time point 5 hours and 10 hours, respectively. The asterisks indicate statistical significant differences of p ≤ 0.05.

This was the case for all strains and the three time points (5, 10, 24 hours), except for the level of IFN-γ following 5 hours stimulation which was not significantly different from mock. The secretion of IL-8 was the highest over the three examined time periods, followed by IL-6 then IL-10, while IFN-γ was induced at a very low level.

Moreover, the levels of IL-8 and IL-6 showed a significant steady increase over time. Conversely, the level of IFN-γ showed only a significant increase between 5 and 10 hours stimulation, but no additional significant enhancement was observed between 10 and 24 hours. Strikingly, the increase in IL-10 over time was steeper than the pro-inflammatory cytokines. Moreover, there were no significant differences in the levels of the inflammatory cytokines between stimulation with LPS and any of the *Campylobacter* strains. Conversely, IL-10 induction by LPS was significantly higher compared to the six *Campylobacter* strains.

### Association between the pro-inflammatory and regulatory cytokines in *Campylobacter*- stimulated PBMC

A significant correlation was found between IL-6 and IL-8 production at all time-points **(Fig 2A)**. Pearson Correlation coefficient was used to examine the correlation between the levels of IL-8 and IL-6 (A), IL-8 and IL-10 (B) and IL-6 and IL-10 (C). Each data point represents stimulation with one strain of *Campylobacter*. P values ≤ 0.05 were considered significant. This was stronger after 5 hours (0.89) stimulation compared to 10 (0.80) and 24 (0.73) hours. Interestingly, the correlation between IL-8 and IL-10 was significant with marked increase over time (0.74, 0.78, and 0.85) **(Fig 2B)**. The correlation between IL-6 and IL-10 was lower than that between IL-8 and IL-10 **(Fig 2C)**. There was no correlation between IFN-γ and any of the other cytokines (data not shown).

## Discussion

The human immune system consists of a network of multiple immune-regulatory control elements that coordinate the immune response elicited by infectious microbes [13]. So far, no studies have been undertaken to analyse whether the immune response against *Campylobacter* in humans is controlled by a balance between pro-inflammatory and regulatory cytokines. Therefore, the aim of the present study was to examine the primary systemic immune response of the two medically important *Campylobacter* species *C*. *jeuni and C*. *coli* on PBMC from healthy individuals. Our results show significant effect of persons on both pro-inflammatory (IL-8, IL-6, IFN-γ) and regulatory (IL-10) cytokines in response to stimulation with *Campylobacter*. This might be linked to individual variations in the constitutive expression levels of toll-like receptors (TLR) [23] which recognize pathogen associated molecular patterns (PAMPs) and generate signals that promote an immune response [13]. Consistent with this explanation, we found that the production of pro-inflammatory (IL-8, IL-6, IFN-γ) cytokines by LPS is significantly influenced by persons (S1 **Table**).

An inter-strain difference in the cytokine production was also observed but less pronounced than the inter-person difference even though the strains used in this study were of different species, different genotypes and either human or chicken isolates. A stronger effect of the strains was observed mainly in IL-10 production, which might be due to variations between the *Campylobacter* strains in the O-linked glycosylation structure of flagella that engage siglec-10 receptors leading to positive regulation of IL-10 [24]. Siglec-10 was found to be expressed on monocytes, minor population of natural killer cells [25] as well as macrophages and monocyte derived dendritic cells [24].

It has been reported previously that the interaction of *Campylobacter* with human intestinal epithelial cells increases IL-8 [10–12] and IL-6 secretion [14]. Here, we found that the secretion of IL-8 was the highest over the three time periods (5, 10 and 24 hours), followed by IL-6 then IL-10, while IFN-γ was induced at a very low level. The peak levels of IL-8 and IL-6 are consistent with studies on clinical human cases of campylobacteriosis [7, 12]. This points to the importance of these two cytokines in the defence against infection with *Campylobacter*. IL-8 can be produced by any cells with TLRs that are involved in the innate immune response. Among them, macrophages are the first cells to release IL-8 in order to recruit other cells. While neutrophils are the primary target cells of IL-8, there is a relatively wide range of cells like endothelial cells, macrophages and others that respond to this cytokine [13]. Moreover, IL-6 is secreted by macrophages, T cells and endothelial cells to stimulate immune response during infection leading to synthesis of a variety of inflammatory mediators including neutrophils [13]. The involvement of neutrophils in host defence against *C*. *jejuni* and *C*. *coli* has been shown [26].

Interestingly, a significant steady increase over time was observed for IL-8 and IL-6, while, the increase in IFN-γ did not show a significant enhancement between 10 and 24 hours. This finding together with the lower production of IFN-γ by PBMC from all donors compared to IL-8 and IL-6 might be attributed to using different campylobacter strains than in other studies which showed a critical role of IFN-γ in immune response to campylobacter infection [16, 27].

Another explanation could be that the donors have been previously experienced *Campylobacter* infection. This is consistent with the findings that IFN-γ production is more robust after the first exposure to campylobacter than subsequent exposures [28]. The low level of IFN-γ coincide with an increase in IL-10. While, the levels of IFN-γ and IL-10 were similar at 5 hours, IL-10 showed a significant up-regulation at 10 and 24 hours post stimulation and hence exceeding the level of IFN-γ. This might explain the low level of IFN-γ as IL-10 is known to downregulate the production of IFN-γ [29].

The increase in IL-10 over time was steeper than the pro-inflammatory cytokines, suggesting that the increase in IL-8 and IL-6 observed up to 10 hours post-exposure results in an elevation of IL-10. A strong correlation was found between IL-8 and IL-6 at all time-points, but it shows a decrease over time. Conversely, the correlation between IL-8 and IL-10 increased over time. The correlation between IL-6 and IL-10 was significant but lower than that between IL-8 and IL-10 and shows a decrease over time. This indicates that *Campylobacter* is capable of inducing an early strong pro-inflammatory response in human immune cells which is then shifted gradually towards a regulatory immune response, most probably to control the damaging inflammation. This is in accordance with *Klancnik et al*. [15] who found beside the high level of anti-inflammatory cytokines, a pronounced increase in IL-10 in the plasma between day 1 and 8 post infection in mice infected with *C*. *coli*. Moreover, *Campylobacter* may benefit from IL-10 production as this might allow the bacteria to persist longer. In chickens, breeds that produce higher levels of IL-10 following *Campylobacter* colonization act as asymptomatic carrier compared to breeds with low IL-10 production [30]. Consistent with other studies [8, 9, 16], levels of cytokines produced by the examined *campylobacter* strains were similar to that occurred in response to stimulation by LPS **(S1 Fig)**. This suggests a role of lipooligosaccharides in initiating an immune response upon infection with *campylobacter* species.

In summary, we found that *C*. *jejuni* and *C*. *coli* elicit an early strong pro-inflammatory response mediated by IL-6 and IL-8. This was associated with a late anti-inflammatory response represented by IL-10 production. The production of inflammatory cytokines was significantly influenced by individual variations rather than by the *Campylobacter* strains. Strikingly, the IL-10 induction was influenced by both the bacterial strain and a person-strain interaction, which might play an important role in reducing the severity of symptoms after infection and the time of shedding after resolution of symptoms. This corroborates our hypothesis that campylobacteriosis is not only driven by bacterial virulence, but rather by the interaction between strain and host immune system, suggesting that the individual differences in the immune reaction might influence the severity of the disease. However, the small number of human subjects involved might not be able to fully reveal the variability of individual responses. A limitation of the present study is the lack of data on the mechanism used by campylobacter to induce stimulation of cytokines as well as the type of immune cells that produce these cytokines. Therefore, further study is required to determine the campylobacter molecules contribute to immune stimulation as well as the overall spectrum of cytokines induced and their cellular sources.

## Supporting information

S1 Fig Correlation between the levels of IL-8, IL-6 and IL-10 following stimulation with the six strains of *Campylobacter* and LPS using Pearson Correlation coefficient. Each data point represents mean value stimulation of one person with the six strains of *Campylobacter* or LPS. P values ≤ 0.05 were considered significant.
(TIF)

S1 Table Effect of person on cytokine production by LPS using One Way ANOVA. (DOCX)
