# Supplementary figures and images for: Temporal induction of pro-inflammatory and regulatory cytokines in human peripheral blood mononuclear cells by Campylobacter jejuni and Campylobacter coli
Source: PLoS One. 2017 Feb 14;12(2):e0171350. doi: 10.1371/journal.pone.0171350 (PMC5308851; doi:10.1371/journal.pone.0171350)

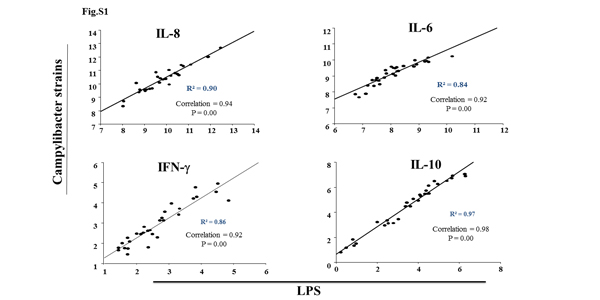

Supplement: S1 Fig — Each data point represents mean value stimulation of one person with the six strains of Campylobacter or LPS. P values ≤ 0.05 were considered significant. (TIF) [file pone.0171350.s001.tif]
