# Supplementary material for: Temporal induction of pro-inflammatory and regulatory cytokines in human peripheral blood mononuclear cells by Campylobacter jejuni and Campylobacter coli
Source: PLoS One. 2017 Feb 14;12(2):e0171350. doi: 10.1371/journal.pone.0171350 (PMC5308851; doi:10.1371/journal.pone.0171350)
Supplement: S1 Table — (DOCX) [file pone.0171350.s002.docx]

**Table S1: Effect of person on cytokine production by LPS using One Way ANOVA**

| Cytokine | Effect of person |
| --- | --- |
| IL-8 | **Sig (0.000)** |
| IL-6 | **Sig (0.004)** |
| IFN-γ | **Sig (0.000)** |
| IL-10 | n. sig (0.761) |
